# Binary polypeptide system for permanent and oriented protein immobilization

**DOI:** 10.1186/1477-3155-8-9

**Published:** 2010-05-12

**Authors:** Enrico Ferrari, Frédéric Darios, Fan Zhang, Dhevahi Niranjan, Julian Bailes, Mikhail Soloviev, Bazbek Davletov

**Affiliations:** 1 MRC Laboratory of Molecular Biology, Cambridge, Hills Road, CB2 0QH, UK; 2 School of Biological Sciences, Royal Holloway University of London, Egham, Surrey, TW20 0EX, UK

## Abstract

**Background:**

Many techniques in molecular biology, clinical diagnostics and biotechnology rely on binary affinity tags. The existing tags are based on either small molecules (e.g., biotin/streptavidin or glutathione/GST) or peptide tags (FLAG, Myc, HA, Strep-tag and His-tag). Among these, the biotin-streptavidin system is most popular due to the nearly irreversible interaction of biotin with the tetrameric protein, streptavidin. The major drawback of the stable biotin-streptavidin system, however, is that neither of the two tags can be added to a protein of interest via recombinant means (except for the Strep-tag case) leading to the requirement for chemical coupling.

**Results:**

Here we report a new immobilization system which utilizes two monomeric polypeptides which self-assemble to produce non-covalent yet nearly irreversible complex which is stable in strong detergents, chaotropic agents, as well as in acids and alkali. Our system is based on the core region of the tetra-helical bundle known as the SNARE (soluble N-ethylmaleimide-sensitive factor attachment protein receptor) complex. This irreversible protein attachment system (IPAS) uses either a shortened syntaxin helix and fused SNAP25-synaptobrevin or a fused syntaxin-synaptobrevin and SNAP25 allowing a two-component system suitable for recombinant protein tagging, capture and immobilization. We also show that IPAS is suitable for use with traditional beads and chromatography, planar surfaces and Biacore, gold nanoparticles and for protein-protein interaction in solution.

**Conclusions:**

IPAS offers an alternative to chemical cross-linking, streptavidin-biotin system and to traditional peptide affinity tags and can be used for a wide range of applications in nanotechnology and molecular sciences.

## Background

Two-component affinity-based tools underlie basic molecular research and are invaluable for the development of drugs and diagnostics [1]. Applications include affinity chromatography, microarray technologies, microplate-based screens and many biotechnological processes [2]. The main factor underlying a successful outcome often relies on firm, irreversible immobilization of a protein in a defined orientation either on a solid surface or in a 3-dimensional matrix. Existing immobilization technologies suffer from a number of disadvantages. For example, in the case of chemical protein coupling [3], one can achieve irreversible surface immobilization, but the product may be in a non-functional state due to orientation issues and chemical modifications. Chemical crosslinking through reactive amino acid side chains of proteins often results in a range of products due to the availability of large number of such groups on a single protein molecule and limited specificity of reactions. The outcome of chemical labelling will depend strongly on reaction conditions such as pH, temperature, etc., and the efficiency of chemical derivatization would often vary from batch to batch. Other chemoselective methods, independent of the reactive terminal amino acids, such as Staudinger ligation [3], require the presence of groups which do not occur in natural or recombinantly produced proteins such as triaryl phosphines and azides. Thus, none of the chemical modification techniques when applied to proteins can achieve the same specificity and selectivity of labelling as affinity-based systems. The most popular binary affinity system utilizes a uniquely strong biotin-streptavidin interaction, however attachment of either biotin or streptavidin (normally tetrameric) to a target protein still requires chemical conjugation and is therefore less site-specific. Recombinant technologies for protein expression, on the other hand, allow a convenient encoding, in the expression vector, of polypeptide affinity tags allowing immobilization on a specific binding substrate. Examples of such polypeptide tag systems include: His-tag binding to metal, glutathione-S-transferase binding to glutathione, maltose-binding protein binding to maltose, strep-tag peptide binding to streptavidin, myc-tag peptide binding to anti-myc antibody-containing surfaces [4-8]. Although it is possible to immobilize a protein in a site-selective way using these polypeptide tags, in all these cases immobilization is either non-permanent or too expensive (antibody-based affinity surfaces). Clearly, the ideal immobilization technique should be capable of both an irreversible coupling as with chemical modifications and selective labelling as affinity based systems. Such system should also allow for a site-specific orientation of the target protein, and be simple, robust and affordable (unlike antibody-based systems, which are prone to degradation, denaturation and are expensive to produce).

Most current affinity tags can only operate in mild conditions, i.e. neutral pH, low ionic strength and physiological temperatures. In the emerging field of nanobiotechnology, conjugation which can resist harsh conditions may be required during fabrication of micro- or nano-arrays, micro-fluidic devices or bio-conjugation to quantum dots or other nanoparticles. Furthermore, enzymes resistant to denaturants, acidic or alkaline conditions are catching attention due to their ability to accelerate reactions in the food and paper industry and in toxic waste removal. Clearly, to better exploit the potential of recombinant proteins for nanobiotechnology, new robust affinity system(s) capable of irreversible capture and immobilization in harsh environments need to be developed. We and others shown previously that three neuronal SNARE proteins, syntaxin, SNAP25 and synaptobrevin, form a very tight tetra-helical bundle commonly known as the SNARE complex [9-12]. In this complex, both syntaxin and synaptobrevin contribute a single α-helix, whereas SNAP25 contributes two α-helices. One fascinating feature of the neuronal SNARE complex is its stability and resistance to harsh treatments, including urea and sodium dodecyl sulphate (SDS) [13]. Only boiling in SDS can break the SNARE complex in vitro; in vivo the complex is dissociated by an intracellular ATPase [14]. Previously, Rothman and colleagues demonstrated that SNARE proteins expressed on the cell surface can fuse cells [15]. The unique properties of the SNARE coiled-coil bundle, however, have not been considered for other applications. Here we report a binary SNARE-based affinity system for protein capture and immobilization, which is permanent and irreversible under physiological buffer conditions.

## Results

**Figure 1:**
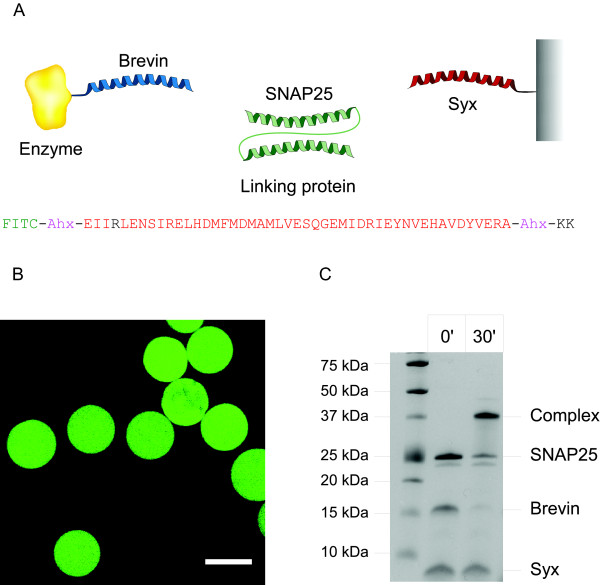
**Syntaxin peptide can be immobilized on solid support and can form the SNARE complex**. (**A**) Schematic showing the immobilization strategy. A fusion containing protein of interest (e.g. enzyme) and brevin can be produced by recombinant means. SNAP25, a two-helical protein, can link brevin and syntaxin into a stable tetra-helical bundle. In the sequence of syntaxin peptide, the fluorescein group (FITC) is linked to the N-terminal glutamate via aminohexaenoic acid (Ahx). The native lysine 204 is replaced by arginine (black) allowing cross-linking to solid support only through the newly introduced C-terminal lysines. (**B**) Image of syntaxin fluorescent beads obtained on a confocal microscope. Scale bar is 50 μM. (**C**) SDS-PAGE Coomassie-stained gel showing that SNAP25, brevin and the syntaxin peptide assemble into a SDS-resistant complex in a 30 min reaction. Molecular weights are indicated on the left.

We first tested whether it is possible to produce a functional SNARE-based immobilization matrix. We synthesized a 47 aa peptide corresponding to the SNARE interaction part of the syntaxin sequence (aa 201-248). The N-terminus of the syntaxin peptide carries fluorescein isothiocyanate (FITC) to aid visualization, while the C-terminus carries two lysines for coupling purposes (Fig. 1A). The internal lysine 204 was replaced by arginine allowing coupling of the peptide to activated BrCN-Sepharose beads only via the introduced lysines. Following the 2 hour coupling reaction, the beads were washed and analysed on a fluorescence microscope. Fig. 1B shows that the fluorescent peptide was successfully attached to beads. In parallel, we tested whether the relatively short syntaxin peptide is capable of forming the SNARE complex. We incubated the syntaxin peptide in the presence of the cytosolic part of synaptobrevin (aa 1-96, brevin for brevity) and full-length SNAP25 (aa 1-206) for 30 minutes at 20°C and analyzed the complex on an SDS-PAGE gel. Fig. 1C shows that the modified 47 aa syntaxin peptide could form an SDS-resistant complex with its corresponding partners. The complex migrates lower than expected from the sum of the three individual components (the complex should be about 40 kDa from the sum of ~6 kDa, ~11 kDa and ~23 kDa for syntaxin peptide, synaptobrevin and SNAP25 respectively and it appears to be ~37 kDa instead). This may be due to the closed conformation of the four-helical bundle which is resistant to SDS. On the other hand individual SNAREs may have an apparent migration higher than their molecular weight as suggested from the apparent size of synaptobrevin and SNAP25 in this SDS-PAGE gel.

**Figure 2:**
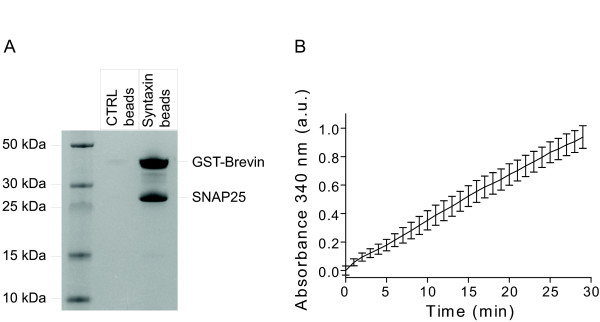
**Immobilization of glutathione-S-transferase (GST) on syntaxin beads**. (**A**) Coomassie-stained gel showing that the GST-brevin fusion protein binds to the syntaxin beads, but not control beads. Binding of GST-brevin occurs via the SNARE complex, as indicated by the presence of SNAP25. (**B**) Graph showing kinetics of the specific GST activity attached to syntaxin beads measured by the increase in absorbance at 340 nm due to conjugation of glutathione to 1-chloro-2,4-dinitrobenzene. The data show mean +/- standard deviation, n = 3.

**Figure 3:**
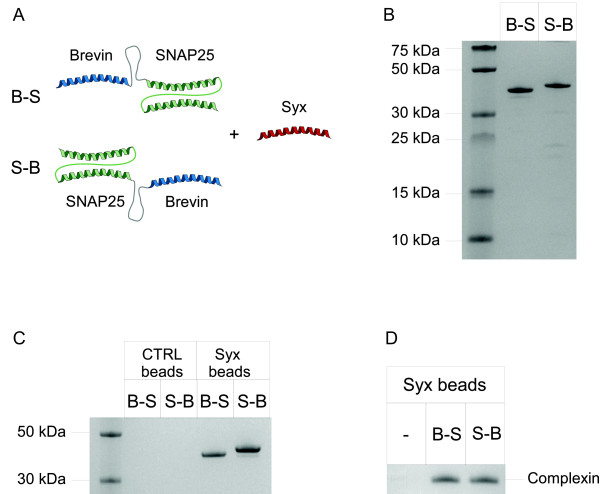
**Three-helical SNARE proteins offer binary immobilization system**. (**A**) Schematic showing fusions of brevin to the N-terminus (B-S) or C-terminus (S-B) of SNAP25. These two proteins are designed to bind syntaxin. (**B**) Coomassie-stained gel showing bacterially-expressed three-helical SNARE proteins. (**C**) Coomassie-stained gel showing that the three-helical SNARE proteins can bind to syntaxin but not control beads. (**D**) Pull-down showing complexin only binds syntaxin beads with B-S or S-B immobilized. Coomassie-stained gel.

To probe SNARE-based immobilization of an example target protein on the syntaxin beads, we used a fusion protein consisting of glutathione-S-transferase (GST) and brevin. We incubated GST-brevin with syntaxin or control beads in the presence of SNAP25 and, following extensive washing of the beads, analyzed bound proteins by SDS-PAGE. For analysis of individual proteins, the beads were boiled in an SDS-containing sample buffer to disrupt the SNARE complex. Fig. 2A shows that GST-brevin bound to the syntaxin beads together with SNAP25; no such binding was observed in the case of control beads. We tested the functionality of bound GST using a colorimetric assay which detects conjugation of glutathione to 1-chloro-2,4-dinitrobenzene. Fig. 2B shows that GST-immobilized on syntaxin beads was functional as measured by the increasing absorbance at 340 nm in a microplate reader. The above tripartite capture system utilizes syntaxin beads, SNAP25 and brevin which can be fused to any desired protein. Most popular affinity systems, however, are of binary nature [2] and therefore we set to simplify the SNARE interaction paradigm by fusing brevin either on N- or C-terminus of SNAP25 (called B-S and S-B, respectively; Fig. 3A). Both proteins were expressed and their purity was analysed on an SDS-PAGE gel (Fig. 3B). The expected size of both B-S and S-B is ~32 kDa, however they migrate much slower in SDS gel (S-B especially). This may be due to a peculiar conformation in the presence of SDS in the running buffer. On the other hand, the complex formed by either B-S or S-B and the syntaxin peptide migrates lower than the single three-helical molecule (data not shown).

When the two proteins were separately mixed with the syntaxin beads we detected binding of each protein (Fig. 3C). To confirm that binding of syntaxin to either B-S or S-B results in the conventional SNARE complex, we tested whether the syntaxin beads with immobilized B-S or S-B can also pull-down complexin, which is known to bind selectively to the neuronal SNARE complex [16]. Indeed, the pull-down in Fig. 3D shows that complexin could specifically bind to syntaxin beads only after addition of B-S or S-B. The complexin binding suggests that the four helices bundle is parallel. Furthermore, the melting temperature of the B-S and S-B complexes, measured by heating in presence of 2% SDS at different temperatures, is 50°C (data not shown), and suggests a tight assembly of SNARE helices [17].

**Figure 4:**
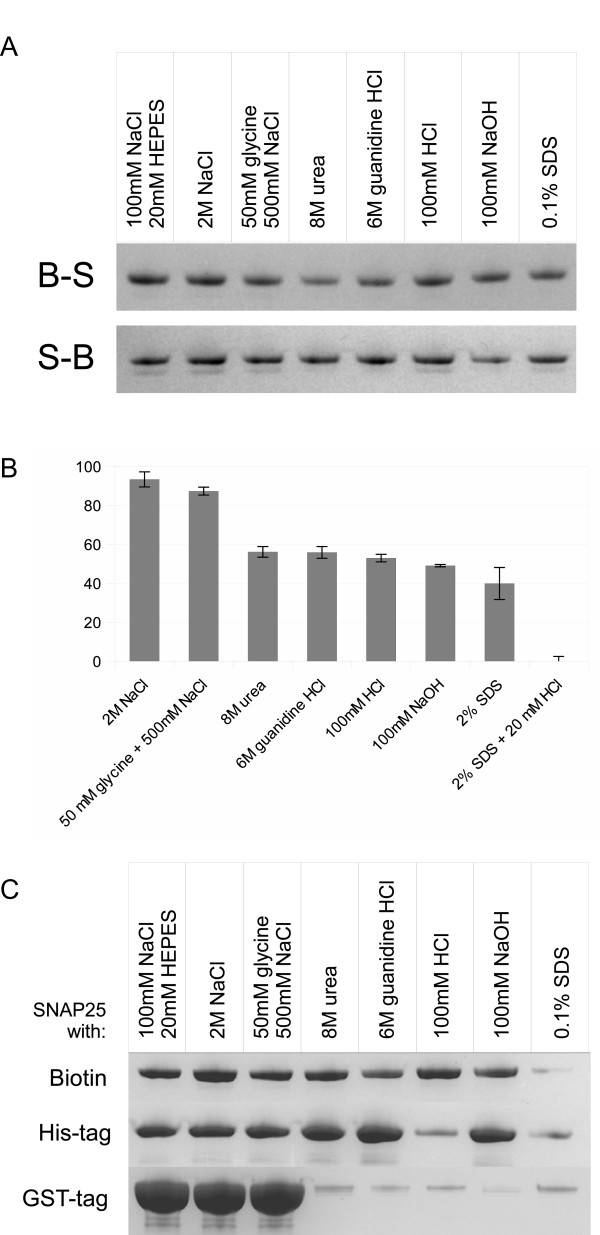
**Resistance of affinity tags to disrupting agents**. (**A**) Coomassie-stained gels showing retention of the three-helical SNARE proteins on syntaxin beads following washes with the indicated eluants. (**B**) A bar chart showing residual amouts of the S-B protein on the syntaxin Biacore chip following application of indicated solutions. The signals were normalized to the original bound S-B protein after the surface plasmon resonance experiment. The data show mean +/- standard deviation, n = 3. (**C**) Coomassie-stained gels showing retention of biotinilated GST-SNAP25, His-tag SNAP25 and GST-tag SNAP25 on streptavidin, Ni-NTA and glutathione beads respectively following washes with the indicated eluants.

Next we probed whether B-S and S-B can be retained on syntaxin beads following washes in harsh conditions. Retainement of both proteins on syntaxin beads was evident even following washes with acidic, alkali or chaotropic reagents (Fig. 4A). Further, we immobilized the syntaxin peptide on the Biacore CM5 chip and tested binding of the S-B protein. Quantification by surface plasmon resonance demonstrated that as much as 50% of originally bound S-B protein is resistant to the harsh treatments used (Fig. 4B). We then performed pull-down assays similar to the one shown in Fig. 4A but using streptavidin beads, nickel-nitrilotriacetic acid (Ni-NTA) beads and gluthatione beads to bind biotinilated-, His-tag- and GST-tag-SNAP25 respectively. Compared to our IPAS, all the three systems fail to retain the bound protein in at least one condition. Biotin/streptavidin shows a very strong binding which can be disrupted by SDS at room temperature, while His-tag can be also eluted by acidic buffer. GST-tag binds very efficiently to the glutathione matrix but then it is easily eluted by detergents, chaotropic agents, as well as by acids and alkali. These results show that the IPAS system is superior to current affinity reagents in terms of resistance to harsh treatments.

**Figure 5:**
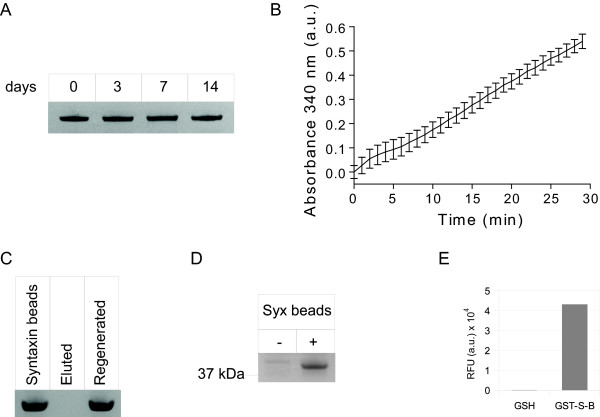
**Immobilization of GST-S-B fusion on syntaxin beads**. (**A**) Coomassie-stained gel showing retention of the recombinant GST-S-B fusion on syntaxin beads at indicated times. (**B**) Graph showing activity of GST-S-B attached to syntaxin beads measured by the increase in absorbance at 340 nm due to conjugation of glutathione to 1-chloro-2,4-dinitrobenzene. The data show mean +/- standard deviation, n = 3. (**C**) Coomassie-stained gel showing that syntaxin beads can be regenerated following a wash with 2% SDS, 20 mM HCl for binding of the S-B three-helical protein. (**D**) The ability of syntaxin beads to bind S-B in presence of calf serum is shown in this pull-down experiment. Coomassie-stained gel. (**F**) Specific binding of the FITC labelled syntaxin peptide to glutathione beads with GST-S-B immobilized in presence of calf serum.

To test the potential of S-B for functional protein immobilization, we tested binding and functionality of GST-S-B fusion protein. GST-S-B was bound to syntaxin beads and its retention on beads was tested during a 14 day period with regular washes. Fig. 5A shows that the S-B tag allows a long-term immobilization of the fused GST enzyme. Test of the transferase activity of GST-S-B following immobilization on syntaxin beads showed that the enzyme was active as measured by the 1-chloro-2,4-dinitrobenzene assay (Fig. 5B). We then addressed the possibility of regeneration of the syntaxin beads. Despite that the S-B tag binds nearly permanently to syntaxin, we noticed that a combination of 2% SDS and 20 mM HCl disrupts the S-B/syntaxin interaction as measured by surface plasmon resonance (Fig. 4B). We therefore tested whether the SDS/HCl combination allows regeneration of syntaxin beads. Fig. 5C shows that the S-B tag can be fully removed from the syntaxin-Sepharose beads by washing with a solution containing both 2% SDS and 20 mM HCl. Remarkably, following a wash in PBS, these beads were able to bind S-B tag back as avidly as before. The regeneration capability of this affinity system suggests that the syntaxin-based capture can be of importance not only for analytical purposes but also for biotechnological applications. Another important feature that affinity systems should have is the binding specificity even in a complex environment where multiple proteins coexist with the target molecule. To this aim, we performed the pull-down of S-B by syntaxin beads in presence of calf serum. Fig. 5D shows that the syntaxin beads can successfully pull down the S-B protein in a specific manner. In addition, we performed pull-down of the FITC labelled syntaxin peptide by either glutathione beads (GSH) only or GSH beads with immobilized GST-S-B in presence of calf serum. As shown in Fig. 5E, the fluorescent peptide bound to GSH beads only if GST-S-B was previously immobilized.

**Figure 6:**
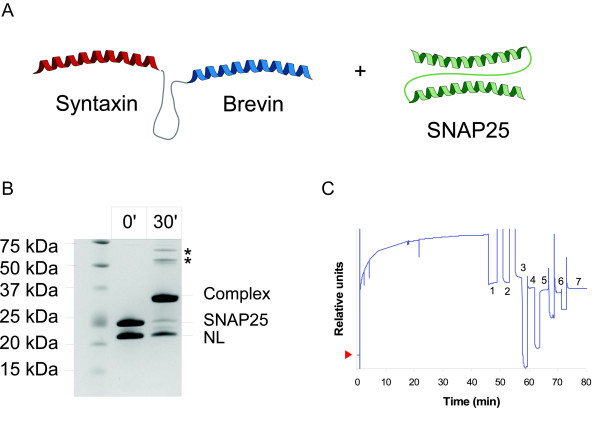
**Two-helical SNARE proteins offer binary immobilization system**. (**A**) Schematic showing fusions of syntaxin to the N-terminus of brevin, referred as NanoLock (NL) in this work. NL is designed to interact with SNAP25. (**B**) Coomassie-stained gel showing that NL and SNAP25 assemble into an SDS-resistant complex in a 30 min reaction. Molecular weights are indicated on the left. The asterisk (*) indicates putative off-pathway oligomeric complexes. (**C**) Surface plasmon resonance sensogram showing the retention of NL on the GST-SNAP25 chip. The red arrow indicates the baseline of GST-SNAP25 crosslinked to the chip surface while (1) shows the level of NL bound to GST-SNAP25 after 45 minutes. A series of washes follows with eluants which are unable to elute the immobilized NL: (2) 2 M NaCl, (3) 50 mM glycine, 500 mM NaCl, (4) 0.1% SDS, (5) 100 mM NaOH, (6) 1% SDS and (7) 100 mM Phosphoric acid.

Although the IPAS system based on a single helix (syntaxin) interacting with a three-helical fusion (S-B or B-S) proved to be effective, we also investigated an alternative binary SNARE configuration made by two two-helical tags. In this affinity system, the first tag is the full length SNAP25 (aa 1-206) and the second is the fusion of syntaxin (aa 195-253) and synaptobrevin (aa 1-84), referred as Nano-Lock (NL) (see the schematic in Fig. 6A). Fig. 6B shows the mixing of these two polypeptides which give a strong SDS-resistant complex. The apparent molecular weight of the complex appears to be lower than the expected sum of the two components perhaps due to the closed conformation of the four-helical bundle in SDS. It has to be noticed that a molecule of SNAP25 can form an SDS resistant complex either with a single molecule of NL or by interacting with the syntaxin part and the synaptobrevin part of two distinct NL molecules, thus generating off-pathway complexes (see Fig. 6B) which are likely to be fibrous assemblies. However, the gel shows that the monomeric complex prevails, perhaps due to kinetic preference. Indeed, by reducing the linker size between the syntaxin and the synaptobrevin SNARE motifs of the NL to a size that doesn't allow a monomeric assembly with SNAP25, we noticed that the binary SDS resistant complex is no longer present, while the oligomeric complexes became enriched at very high molecular weights, suggesting the formation of fibrous assemblies (data not shown).

Similarly to what we did for the syntaxin/three-helical IPAS, we then immobilized GST-SNAP25 on a Biacore chip to prove the possibility of capturing the NL on the chip surface. Fig. 6C shows the effective immobilization of NL on top of SNAP25 and the strong resistance of the complex to a series of harsh washes.

**Figure 7:**
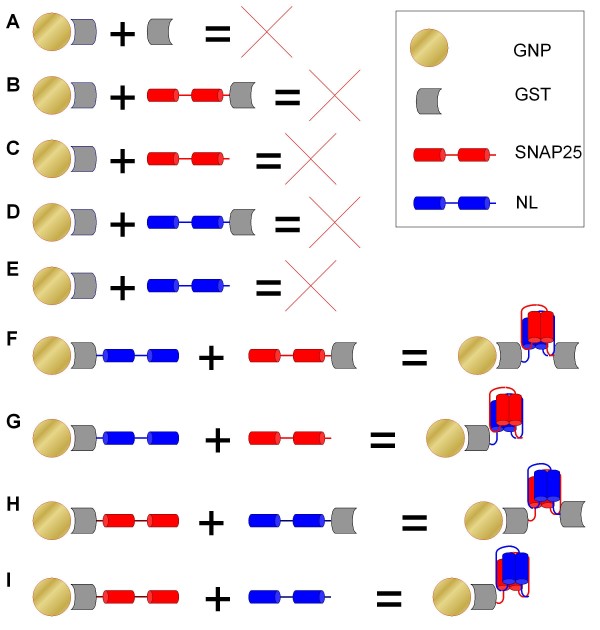
**A scheme showing protein immobilization and capture on gold nanoparticles (GNPs)**. (**A-E)**, GST-derivatised GNPs. (**A**) The addition of extra GST does not result in any detectable interaction. (**B**) The addition of GST-SNAP25 fusion protein does not result in any detectable interaction. (**C) **The addition of SNAP25 does not result in any detectable interaction. (**D**) The addition of GST-NL fusion protein does not result in any detectable interaction. (**E**) The addition of NL fusion peptide does not result in any detectable interaction. (**F-G**) GNPs derivatised with GST-NL fusion protein. (**F**) The addition of GST-SNAP25 fusion protein results in specific interaction and the formation of the tight tetra-helical assembly. (**G**) The addition of SNAP25 construct results in specific interaction and the formation of the tight tetra-helical assembly. (**H-I**) GNPs derivatised with GST-SNAP25 fusion protein. (**H**) The addition of GST-NL fusion protein results in specific interaction and the formation of the tight tetra-helical assembly. (**I**) The addition of NL fusion peptide results in specific interaction and the formation of the tight tetra-helical assembly. In all panels, the filled circle symbolizes a gold nanopartice, a grey-filled arch denotes a GST protein, red coloured cylinders represent the two helices based on the SNAP25 protein sequence, blue coloured cylinders indicate a NL fusion peptide.

**Figure 8:**
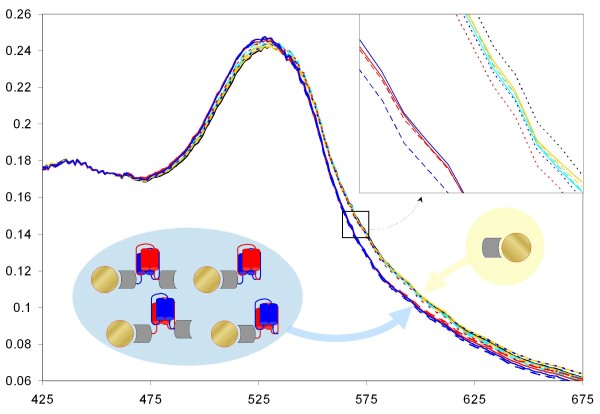
**Absorption spectra of derivatised gold sols reacted with different fusion proteins and constructs**. Blue solid and dashed lines show absorption spectra of GNP-GST-NL derivatised gold sol reacted with GST-SNAP25 and SNAP25 respectively. Red solid and dashed lines show absorption spectra of GNP-GST-SNAP25 derivatised gold sol reacted with GST-NL and NL respectively. Turquoise solid and dashed lines show absorption spectra of GNP-GST derivatised gold sol reacted with GST-SNAP25 and GST-NL respectively. Dark yellow solid and dashed lines show absorption spectra of GNP-GST derivatised gold sol reacted with SNAP25 and NL peptides respectively. Dotted red line show absorption spectrum of the GNP-GST-SNAP25 derivatized gold sol incubated with GST protein alone. Dotted blue line show absorption spectrum of the GNP-GST-NL derivatised gold sol incubated with GST protein alone. Dotted black line show absorption spectrum of the GNP-GST derivatised gold sol incubated with GST protein alone. Schematic images of the derivatised GNPs and the colour coding are the same as in Fig. 7. The insert (top right corner) shows blown up section of the absorption spectra to illustrate the two highly similar groups of GNPs identified.

To further evaluate the usability of the binary peptide capture system we tested protein immobilization and capture on gold nanoparticles (GNPs). We chose to monitor GNP plasmon resonance by measuring absorption of gold sols derivatized and reacted with a set of proteins, including GST, GST-SNAP25, GST-NL, SNAP25 and NL (Fig. 7). We detected interaction between GNP-GST-SNAP25 and GST-NL, and NL alone, but not with GST alone. GNP-GST-NL was found to interact with GST-SNAP25, SNAP25, but not with GST alone (Fig. 8). Gold without any of the binary peptide fragments (GNP-GST) has shown no change in optical properties, proving that none of the GST-SNAP25, SNAP25, GST-NL, NL or GST alone would interact with GNP-GST. Fig. 8 indicates that following the formation of the tetra-helical bundle, the characteristic absorption peak moved towards the shorter wavelengths, apparently indicating more tight protein packing on the GNP surface. Derivatized but non-reacting GNP-GST sols absorption spectra (turquoise and dark yellow lines and the dotted black line in Fig. 8) are not distinguishable from the absorption of GNP-GST-SNAP25 or GNP-GST-NL incubated with GST alone (i.e., no specific protein-protein interaction). The one common feature of these GNPs is that no peptide self-assembly occurred on the surface of these GNPs. All these spectra differ clearly form the spectra of GNP-GST-SNAP25 or GNP-GST-NL incubated and reacted with GST-NL, NL, GST-SNAP25 and SNAP25 (blue solid and dashed, and red solid and dashed lines respectively, Fig. 8). These four spectra are nearly identical to each other, but differ from the spectra measured for GNP-GST derivatized gold, irrespective of the second protein added.

**Figure 9:**
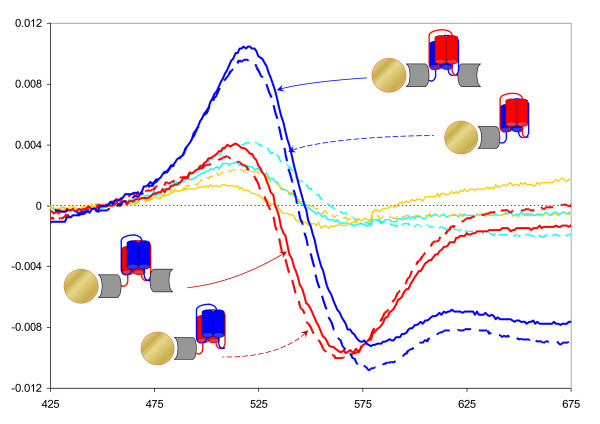
**Difference absorption spectra of derivatised gold sols reveal SPR peak shift following the tetra-helical peptide self-assembly**. Blue solid and dashed lines show difference spectra for GNP-GST-NL derivatized gold sol reacted with GST-SNAP25 and SNAP25 respectively after subtraction of the absorption spectrum measured for the same GNP-GST-NL gold sol incubated with a non-reacting GST protein alone. Red solid and dashed lines show difference spectra for GNP-GST-SNAP25 derivatized gold sol reacted with GST-NL and NL respectively after subtraction of the absorption spectrum measured for the same GNP-GST-SNAP25 gold sol incubated with a non-reacting GST protein alone. Turquoise solid and dashed lines show absorption spectra of GNP-GST derivatized gold sol reacted with GST-SNAP25 and GST-NL respectively after subtraction of the absorption spectrum measured for the same GNP-GST gold sol incubated with GST protein alone. Dark yellow solid and dashed lines show absorption spectra of GNP-GST derivatized gold sol reacted with S25 and NL peptides respectively after subtraction of the absorption spectrum measured for the same GNP-GST gold sol incubated with GST protein alone. Schematic images of the derivatized reacted GNPs and the colour coding are the same as in Fig. 7.

Differential spectra show clear and consistent changes in the spectral properties of GNPs following the formation of the protein complex (Fig. 9). Differential spectra show identical changes for GNP-GST-SNAP25 interacting with either GST-NL or NL alone. Optical properties of the GNP-GST-NL sol changed similarly for both GST-SNAP25 and SNAP25. Fig. 9 indicates that after GNP derivatization, any additional SPR peak shifts depend only on the protein folding rather than on the amount of additional protein immobilized through the protein-protein interaction. Difference spectra for GNP-GST-SNAP25 reacted with either GST-NL or NL alone (solid and dashed blue lines on Fig. 9) are virtually identical to each other, so are the difference spectra for GNP-GST-NL reacted with either GST-SNAP25 or SNAP25 alone (solid and dashed red lines on Fig. 9). These difference spectra are obtained by subtracting absorption spectra obtained for GNP-GST-SNAP25 or GNP-GST-NL (respectively), incubated with GST alone, to compensate for any possible differences in the derivatized gold sol absorption. However Fig. 8 indicates that such differences were minute if at all existed (see nearly identical red and blue dotted lines in Fig. 8). Clear difference between the derivatized GNP-GST-SNAP25 reacted with GST-NL (solid blue line in Fig. 9) and GNP-GST-NL reacted with GST-SNAP25 (solid red line in Fig. 9) indicates that despite the apparently similar overall protein load, the absorption spectra are different. Similar arguments apply to the GNP-GST-SNAP25 reacted with NL peptide alone (dashed blue line in Fig. 9) and GNP-GST-NL reacted with SNAP25 alone (dashed red line in Fig. 9). The main difference between the above pairs is the orientation of the tetra-helical assembly in relation to the GNP surface, rather than protein load. We therefore conclude that our system is sensitive to and might be suitable for determining differences in the orientation of the absorbed proteins.

## Discussion

Here we described a novel binary affinity system for protein capture that can withstand very harsh conditions. The irreversible protein attachment system (IPAS) utilizes 3 SNARE proteins which were converted into two tags. Our affinity system is based on the neuronal SNARE complex, a bundle of four α-helices interacting through strong hydrophobic forces [10]. It is believed that the SNARE complex formation happens by a 'zippering' mechanism starting at the N-termini of four SNARE motifs. The complex has an extremely slow dissociation rate with a half-life estimated to be a billion years under non-denaturating conditions in vitro but can be dissociated inside cells by an ATPase [14,18]. Generally, SNARE proteins play a key role in fusion of intracellular vesicles with their target membranes. To date, more than 100 SNARE proteins have been discovered which carry highly conserved ~70 aa heptad repeat motifs responsible for tight SNARE interactions [19]. It, thus, will be of interest to evaluate usefulness of other SNARE proteins for affinity systems. Tandem fusion of SNARE proteins is a practical invention which has not been considered previously, but as shown here allows production of high-affinity reagents. Naturally, the most attractive feature of the SNARE-based protein capture is the potential of the IPAS tags to be fused to proteins of interest via recombinant means. The resulting fusion products can then be nearly permanently immobilized to a solid support via a simple mixing with the corresponding immobilization support (i.e., syntaxin beads, syntaxin or GST-SNAP25 Biacore chips, GST-SNAP25 or GST-NL gold nanoparticles). When necessary, either of the tags in our binary system can be chemically linked to surfaces of beads, chips and microarray plates, or modified by chemical or recombinant introduction of functional groups. Our tested SNARE-based bimolecular affinity system affords an inexpensive, nearly irreversible linking of required protein modules or firm capture of tagged molecules on surfaces. The irreversible nature of the SNARE complex makes the conventional thermodynamic analysis difficult; under normal buffer conditions the dissociation of the IPAS peptides is not detectable with either of the methods we tested (beads pull down, Biacore) and was impossible to estimate even for naturally occurring SNARE complexes [20]. The use of α-helical bundles as affinity tags has been attempted before based on heterodimerization of coiled-coils ~40 aa peptides [21-23]. However, in contrast to the de novo engineering, we chose a biomimetic strategy focusing on a known tight interaction that was perfected by evolution to drive fusion of cellular membranes [19]. Our work presents the first evidence that an affinity system based on SNARE proteins can work, maintaining the unique property of the SNARE complex - extremely stable interaction that can withstand harsh conditions. Although here we presented two IPAS systems that are based on a single helix (syntaxin) interacting with a three-helical fusion (S-B or B-S) and an alternative IPAS based on two double helices (NL and SNAP25), we anticipate that other SNARE configurations would be also possible.

As a practical application in the field of nanobiotechnology we have reported the assembly of the tetra-helical complex on the surface of gold nanoparticles, detected by measuring the change in the colloidal gold surface plasmon resonance peak. Red shift in the SPR peak of gold nanoparticles depends on and changes linearly with the refractive index of the surrounding medium [24]. The red shift due to the immobilization of protein is also well documented [25,26] and results from the apparent increase in the overall size of the gold nanoparticles. We have observed slight blue shift following the assembly of the tetra-helical "NanoLock" complex. No change in optical properties was detected when any of the non-interacting proteins were incubated with the derivatized gold sol. The blue shift indicates that the assembly is likely to result in the increased density of protein packing on the surface of the gold, which is expected, because of the nature of the binary peptides, based on the virtually irreversible binding of SNARE proteins. The addition of GST protein to the NL peptide apparently makes no difference for the tetra-helical self-assembly of GST-NL or NL with GNP-GST-SNAP25. And neither the addition of GST affects self-assembly of SNAP25 with GNP-GST-NL. This is significant because it means that our self-assembling system is not affected by the protein "load" added to either of the binary peptides (SNAP25 or NL). Our results also show that the self-assembly of SNAP25 and NL peptides may be easily controlled irrespective of the protein "load" used. We have also shown that our system is sensitive to the orientation of proteins on the gold surface. This is consistent with the previously reported ability of GNP based methods to distinguish chiral differences [27,28]. Thus, our results indicate that gold nanoparticles uses are not limited to the detection of protein-protein interactions but may also be used for monitoring protein folding. Previously reported applications of gold nanoparticles for protein conformational changes were limited to detecting pH changes [29,30], thermodynamic stability, unfolding or to aggregation assays. However, unlike previous reports, where protein folding was detected only through nanoparticle aggregation [31-33], the NanoLock binary peptides assembly does not result in the loss of gold nanoparticles, which remain in the sol and could therefore be used for downstream applications.

The emerging field of nanotechnology increases the demand for tailored conjugation methods for the development of nanochips, microarrays and also for nanodevices for drug delivery [34-37]. Biomaterial and tissue engineering can also benefit from the presented conjugation method for decoration of inert fibrous scaffolds with biologically active molecules [38]. Finally, industrial processes involving immobilized enzymes could require non-covalent yet stable conjugation specifically designed to be resistant to harsh treatments [39].

## Conclusions

We designed three pairs of self assembling polypeptides mimicking the neuronal SNARE complex: the first is made by a 6 kDa sytaxin peptide and the 32 kDa fusion of synaptobrevin and SNAP25 (B-S), the second is made by the same syntaxin peptide and the 32 kDa fusion of SNAP25 and synaptobrevin (S-B) and the third pair is represented by the SNAP25 protein and a 17 kDa fusion of syntaxin and brevin. The affinity systems presented here provides a novel concept that can be utilized for tailored applications in many different technologies.

## Methods

### Preparation of polypeptides

GST fusions with the full-length rat SNAP25B (aa 1-206) with cysteine to alanine mutations, rat synaptobrevin2 (aa 1-96), complexin II and GST alone were cloned in pGEX-KG vector. His-tag rat SNAP25B (aa 1-206) with cysteine to alanine mutation was cloned on pET vector. Plasmids encoding S-B and B-S fusion proteins were made by attaching optimized SNAP25B DNA (commercially obtained from ATG Biosynthetics) on the N-terminus and C-terminus of synaptobrevin2 (aa 1-84) in the pGEX-KG vector. The plasmid encoding the NL fusion protein was made by attaching the DNA sequence of rat syntaxin3 (195-253) to rat synaptobrevin2 (1-84) in the pGEX-KG vector. The amino acid sequences of S-B, B-S and NL are:

S-B:

GSADESLESTRRMLQLVEESKDAGIRTLVMLDEQGEQLERIEEGMDQINKDM KEAEKNLTDLGKFAGLAVAPANKLKSSDAYKKAWGNNQDGVVASQPARVV DEREQMAISGGFIRRVTNDARENEMDENLEQVSGIIGNLRHMALDMGNEIDT QNRQIDRIMEKADSNKTRIDEANQRATKMLGSGSGSSGASGEQKLISEEDLSG GSAGSGSSAGMSATAATVPPAAPAGEGGPPAPPPNLTSNRRLQQTQAQVDEV VDIMRVNVDKVLERDQKLSELDDRADALQAGASQFETSAAKL,

B-S:

GSMSATAATVPPAAPAGEGGPPAPPPNLTSNRRLQQTQAQVDEVVDIMRVN VDKVLERDQKLSELDDRADALQAGASQWETSAAKLSGAGSGAGSAGSGSAE DADMRNELEEMQRRADQLADESLESTRRMLQLVEESKDAGIRTLVMLDEQG EQLERIEEGMDQINKDMKEAEKNLTDLGKFAGLAVAPANKLKSSDAYKKAA GNNQDGVVASQPARVVDEREQMAISGGFIRRVTNDARENEMDENLEQVSGII GNLRHMALDMGNEIDTQNRQIDRIMEKADSNKTRIDEANQRATKMLGSG,

NL:

GSEGRHKDIVRLESSIKELHDMFMDIAMLVENQGEMLDNIELNVMHTVDHV EKARDEAKRAGILDSMGRLELKLMSATAATVPPAAPAGEGGPPAPPPNLTSN RRLQQTQAQVDEVVDIMRVNVDKVLERDQKLSELDDRADALQAGASQFETS

AAKL.

GST fusion proteins were produced in BL21 Escherichia coli and purified on glutathione-sepharose beads (Amersham Biosciences), followed either by elution with glutathione (GST-tag proteins) or by thrombin cleavage (SNARE part only). His-tag SNAP25 was purified using Ni-NTA agarose beads (Qiagen) and eluted with Imidazole. Eluted proteins were further purified by gel filtration on a Superdex 200 column (Amersham Biosciences) equilibrated in buffer A (20 mM HEPES, 100 mM NaCl, pH 7.2). The 47 aa syntaxin peptide corresponding to the SNARE interaction part of the syntaxin sequence 201-248 was commercially obtained from Peptide Synthetics. Biotinilation of GST-SNAP25 have been obtained using biotin-maleimide from Sigma.

### Cross-linking of the syntaxin peptide to beads

0.75 mg of syntaxin peptide was cross-linked to 0.28 g (dry weight) of CNBr-activated Sepharose 4B beads (Amersham Biosciences) which were pre-washed in 1 mM HCl and pre-hydrolysed for 4 h at room temperature in the coupling buffer (0.1 M NaHCO3, 0.5 M NaCl, pH 8.3). Coupling reaction have been carried in the same buffer for 2 h at 20°C followed by an overnight blocking of the active groups at 4°C with 1 M ethanolamine, pH 8.5. Beads were washed with 0.1 M acetate buffer, 0.5 M NaCl, pH 4.0, followed by 0.1 M Tris-HCl, 0.5 M NaCl, pH 8.0, and finally buffer A. Fluorescence was visualized on a Bio-Rad confocal microscope (Fig. 1B). Control beads were prepared following the same protocol but in absence of the syntaxin peptide.

### SNARE complex formation

To analyze formation of the SDS-resistant SNARE complex (Fig. 1C and Fig. 6B), proteins were incubated (final concentration 1 μM) in buffer B (20 mM HEPES, 100 mM NaCl, 0.8% (w/v) n-octylglucoside, pH 7.2) for 30 min at 20°C in a total volume of 20 μl. We noticed that n-octylglucoside aids the formation of the SNARE bundle. The reactions were stopped by the addition of SDS-containing sample buffer, and proteins were separated by SDS-PAGE and visualised by Coomassie staining. Note that SNARE complex, likely due to its closed conformation, migrates faster than the apparent sum of the monomers sizes.

### Protein pull-down

Syntaxin and control beads (see preparation above, Fig. 2A, B, 3C, 3D, 4A, 5A, B, C, D), streptavidin-sepharose (Sigma, Fig. 4C), Ni-NTA-agarose (Qiagen, Fig. 4C) and glutathione-sepharose beads (Amersham Biosciences, Fig. 4C, 5E) in buffer B were incubated in the presence of an excess of proteins for 30 min at 20°C in a reaction volume of 50 μl with constant shaking. In the experiments shown in Fig. 5D and 5E buffer B has been replaced by calf serum. The beads were washed three times with 20 mM HEPES, pH 7.0, 1 M NaCl, 1 mM EDTA, 0.1% Triton X-100 and 1 mM DTT by low-speed centrifugation followed by two additional washes with buffer A. When using Ni-NTA beads EDTA was omitted to avoid elution by chelation. Bound protein was eluted into SDS containing sample buffer, heated at 100°C for 3 min and analysed by SDS-PAGE and Coomassie staining. When testing disassembly of the binary affinity system, various solutions indicated in the figures were applied to the beads for 10 min at 20°C followed by standard washes.

### GST activity assay

GST activity assay of the immobilized enzyme was performed with 10 μl beads containing 2 μg of GST-S-B or control beads, according to the manufactures instructions (Sigma). Absorbance at 340 nm was measured in a Tecan plate reader and presented after subtraction of the background signal from control beads.

### Surface Plasmon Resonance measurements

Experiments shown in Fig. 4B and 6C were performed using a Biacore 2000 system (GE Healthcare). Following the initial wash of the CM5 chip with 1% SDS (1 min), 100 mM Phosphoric acid (1 min) and 100 mM NaOH (2 min), the chip was used to covalently immobilise either the syntaxin peptide or GST-SNAP25 (0.05 mg/ml in 0.1 M acetate buffer, pH 5, containing 0.5% DMSO and 0.8% n-octylglucoside. Following blocking of the chip surface with 0.1 M ethanolamine and a wash with 1% SDS (1 min), 100 mM phosphoric acid (1 min) and 100 mM NaOH (2 min), the chip surface was loaded with 0.13 mg/ml S-B protein in buffer B for 5 min (Fig. 4B) and 0.10 mg/ml NL in buffer B for 45 min (Fig. 6C). To check stability of the formed complex, the loaded chip was washed consecutively with a selection of washing or denaturing reagents for 1 min each. To control for any background drifts and for background subtraction the data were compared to the values obtained for the unloaded channel. All measurements were performed at 25°C.

### Gold nanoparticles synthesis and protein adsorption

Gold sols were prepared by reducing Tetrachloroauric acid hydrate with sodium citrate. 40 ml of 0.02% w/w solution of HAuCl4 (Alfa Aesar 36400) in deionised water (equivalent to 0.01% w/w gold) was heated to boiling point under constant stirring. 4 ml of 1% sodium citrate was added under rapid stirring, which continued for another 15 minutes. Sodium azide was added to cooled gold sols to final concentration of 0.05% (w/v). To determine protein binding capacity of gold nanoparticles (GNP), series of bovine serum albumin (BSA) dilutions ranging from 1 μg/ml to 10 mg/ml were made. 100 μl of each of BSA dilutions was added to individual 500 μl aliquots of colloidal gold and the samples were incubated at 25°C with constant, gentle agitation. Following the 30 min incubation 600 μl aliquots of 20% NaCl were added to individual GNP-BSA samples. The GNP-BSA sample with the lowest BSA content (100 μl or 1 mg/ml BSA per 500 μl of the GNP preparation) where no colour change was observed contained sufficient protein for total coverage of the colloidal gold present. The same ratio of protein to GNPs was used in all following experiments.

### NanoLock interaction detection using gold nanoparticles

Three groups of GNPs were produced by derivatization with GST, GST-S25 and GST-NL proteins. Each protein was diluted to 0.02% w/v with buffer A and a number of individual GNP samples for each protein were made by mixing 50 μl of 0.02% w/w GNP sol with 300 μl of GNP buffer (0.1% sodium citrate, 0.02% NaN_3_) and 50 μl of 0.02% GST, GST-S25 or GST-NL to make GNP-GST, GNP-GST-S25 and GNP-GST-NL respectively. Following 70 min incubation at 25°C with gentle agitation, n-octylglucoside was added to each sample to a final concentration of 0.8%. Following 10 min incubation at 25°C a second set of proteins was added to the fully derivatized GNP-protein sols. Protein concentrations were 0.02% w/v for GST, GST-S25 and GST-NL, and 0.01% for S25 and NL proteins. 50 ul of each protein was added, followed by 30 min incubation at 25°C. Absorption spectra were taken using Helios Alpha UV-Vis spectrophotometer, wavelength resolution 1 nm. All samples were prepared individually at least in duplicate and the experiment repeated twice.

## Competing interests

The authors declare that they have no competing interests.

## Authors' contributions

EF carried out most of the experiments, participated in the design of the study and drafted the manuscript. FD engineered the self-assembling peptides and fusion constructs and participated in the design of the overall study. FZ and DN participated in the design of the engineered proteins by optimizing the expression and purification of the new recombinant fusion proteins. JB carried out nanoparticles synthesis and protein immobilization experiments. MS participated in the design of the study and contributed to the drafting of the manuscript. BD conceived, coordinated the study and contributed to the drafting of the manuscript. All authors read and approved the final manuscript.
